# The Prognostic Value of Decreased LKB1 in Solid Tumors: A Meta-Analysis

**DOI:** 10.1371/journal.pone.0152674

**Published:** 2016-04-01

**Authors:** Jian Xiao, Yong Zou, Xi Chen, Ying Gao, Mingxuan Xie, Xiaoxiao Lu, Wei Li, Bixiu He, Shuya He, Shaojin You, Qiong Chen

**Affiliations:** 1 Department of Geriatrics, Respiratory Medicine, Xiangya Hospital of Central South University, Changsha, China; 2 Department of Respiratory Medicine, Xiangya Hospital of Central South University, Changsha, China; 3 Department of Biochemistry and Biology, University of South China, Hengyang, China; 4 Laboratory of Cancer Experimental Therapy, Atlanta Research & Educational Foundation (151F), Atlanta VA Medical Center, Decatur, GA, United States of America; University Medical Center of Princeton/Rutgers Robert Wood Johnson Medical School, UNITED STATES

## Abstract

**Background:**

Liver kinase B1 (LKB1) is a protein kinase that regulates the growth, integrity and polarity of mammalian cells. Recent studies have reported the prognostic value of decreased LKB1 expression in different tumors. However, the results of these studies remain controversial. Therefore, this meta-analysis was performed to more accurately estimate the role of decreased LKB1 in the prognostication of human solid tumors.

**Methods:**

A systematic literature search in the electronic databases PubMed, Embase, Web of Science and CNKI (updated to October 15, 2015) was performed to identify eligible studies. The overall survival (OS), relapse-free survival (RFS), disease-free survival (DFS) and clinicopathological features data were collected from these studies. The hazard ratios (HRs), odds ratios (ORs) and 95% confidence intervals (CIs) were calculated and pooled with a random-effects models using Stata12.0 software.

**Results:**

A total of 14 studies covering 1915 patients with solid tumors were included in this meta-analysis. Decreased LKB1 was associated with poorer OS in both the univariate (HR: 1.86, 95%CI: 1.42–2.42, P<0.001) and multivariate (HR: 1.55, 95%CI: 1.09–2.21, P = 0.015) analyses. A subgroup analysis revealed that the associations between decreased LKB1 and poor OS were significant within the Asian region (HR 2.18, 95%CI: 1.66–2.86, P<0.001) and obvious for lung cancer (HR: 2.16, 95%CI: 1.47–3.18, P<0.001). However, the articles that involved analyses of both RFS and DFS numbered only 3, and no statistically significant correlations of decreased LKB1 with RFS or DFS were observed in this study. Additionally, the pooled odds ratios (ORs) indicated that decreased LKB1 was associated with larger tumor size (OR: 1.60, 95%CI: 1.09–2.36, P = 0.017), lymph node metastasis (OR: 2.41, 95%CI: 1.53–3.78, P<0.001) and a higher TNM stage (OR: 3.35, 95%CI: 2.20–5.09, P<0.001).

**Conclusion:**

These results suggest that decreased LKB1 expression in patients with solid tumors might be related to poor prognosis and serve as a potential predictive marker of poor clinicopathological prognostic factors. Additional studies are required to verify the clinical utility of decreased LKB1 in solid tumors.

## Introduction

Liver kinase B1 (LKB1) is a protein kinase also known as serine/threonine kinase 11 that is encoded by the STK11 gene in humans [1]. LKB1 is the homologue of par-4 in non-mammalian species [2] and can regulate early embryonic development in both mammals and non-mammals [2–4]. LKB1 has been linked to the regulation of epithelial integrity and polarity [5,6]. The loss of LKB1 disrupts epithelial cell polarity and promotes cancer progression, invasion and metastasis [7,8]. Experimental evidence also indicates that LKB1 deficiency can cause adenocarcinomas to transdifferentiate into squamous cell carcinomas [9]. Therefore, LKB1 is considered a tumor suppressor kinase [10].

Studies have demonstrated that low LKB1 protein expression is associated with worse overall survival (OS) in human breast cancer [11]. Additionally, low LKB1 expression levels in human pancreatic ductal adenocarcinomas and decreased expression of LKB1 in hepatocellular carcinoma patients are poor prognostic factors [12,13]. Reports continue to suggest that LKB1 loss at the protein level plays a role in the poor outcomes of patients with colorectal cancer and non-small cell lung cancer [14,15]. Moreover, studies have also indicated that low expression of LKB1 is associated with tumor clinicopathological features [15,16].

Although some evidence suggests that decreased LKB1 is an important factor that is implicated in poorer survival in solid tumor patients [11–16], some conflicting results have also been reported [17,18]. However, these results still seem to be controversial. Consequently, we initiated a meta-analysis to determine the significance of decreased LKB1 expression in the prediction of clinical outcomes and to examine the association between decreased LKB1 and the clinicopathological parameters of solid tumors.

## Materials and Methods

### Literature Search Strategy

The literature relevant to LKB1 expression and survival in solid tumors was searched in the PubMed, Embase, Web of Science and China National Knowledge Infrastructure (CNKI) databases through October 15, 2015. The search terms included the following key words in various combinations: LKB1, STK11, liver kinase b1, prognosis, prognostic, survival, and overall survival. The list of publications was limited to human studies and restricted to those published in Chinese or English. The references of the review articles and primary research were further searched to identify additional potentially relevant studies to avoid omission due to the electronic search approach.

### Study Inclusion and Exclusion Criteria

The studies that were included in this meta-analysis met the following criteria: (1) a pathological diagnosis of cancer was made;(2) original published studies with full text that measured LKB1 protein expression in patients with any type of tumor via immunohistochemistry or western blotting; (3) associations of LKB1 expression with overall survival (OS), relapse-free survival (RFS), disease-free survival (DFS), or clinicopathological features were described; (4) hazard ratios (HRs) and 95% confidence intervals (CIs) were reported or could be calculated based on the information in the paper; and (5) when the same author reported repeated results from the same population, the most complete report was included.

The exclusion criteria for this meta-analysis were as follows: (1) unpublished papers; (2) laboratory articles, review and letters; (3) articles with only animal experiments; and (4) studies without information about survival outcomes or survival curves and those in languages other than Chinese and English.

### Quality Assessment

Two independent reviewers (Xi Chen and Xiaoxiao Lu) scored the qualities of the selected papers using the Newcastle—Ottawa Quality Assessment Scale (NOS), which was referenced in a previously published paper [19] (S1 Table). Briefly, the score of each paper was decided based on selection, comparability and outcome according to the NOS. Each appraised study received a score between 0 and 9. NOS scores of 9–7, 6–4 and 3–1 were defined as high-, intermediate- and low-quality studies, respectively. Discrepancies were discussed until a consensus was reached regarding the final score for each paper.

### Data Extraction

For the eligible studies, two investigators (Ying Gao and Wei Li) independently extracted the following data: first author’s name, publication year, region, type of cancer, number of patients, patients’ sexes and ages, follow-up times, test methods, staining positions, cut-off values, survival data (including OS, RFS or DFS), analysis method, and clinicopathological parameters, such as tumor differentiation, tumor size/invasion depth, lymph node metastasis and TNM stage. For studies that presented only Kaplan-Meier curves, Engauge Digitizer version 4.1 (http://digitizer.sourceforge.net/, a free down-loaded software) was used to extract the survival data. The estimates of the HRs and 95% CIs were calculated by Tierney’s method as previously described [20]. Subsequently, the raw data were entered into GraphPad Prism 6.0 (GraphPad Software, Inc.) to produce Kaplan-Meier curves for comparison with the published curves [21]. Any disagreements were adjudicated by discussion until a consensus was reached.

### Statistical Analysis

This meta-analysis was performed using Stata 12.0 (Stata Corporation, College Station, TX, USA) software. Generic inverse variance weighting was used to pool the HRs. When the result of a Q-test (I^2^>50% or P<0.05) indicated heterogeneity between the studies, the random-effects model was used for the meta-analysis. Otherwise, a fixed-effects model was used [22]. An HR greater than 1 indicated poor prognosis in patients with decreased LKB1. The chi-squared test (Cochrane’s Q test) and I-squared statistical test were used to analyze the heterogeneity between studies. A sensitivity analysis was used to test the influences of individual studies on the pooled HR to evaluate the stability of the meta-analysis. Because unequal characteristics might have been included in the eligible studies, subgroup stratification analyses were performed according to the testing method, region, cancer type, staining position and analysis method to identify the sources of heterogeneity. Funnel plots were used to graphically represent the publication bias. Begg’s (rank correlation) and Egger’s (regression asymmetry) tests were adopted to confirm the publication bias.

Pooled estimates of the odds ratios (OR) were calculated using the Mantel-Haenszel method to estimate the correlations of LKB1 expression with the clinicopathological parameters, which included tumor differentiation, tumor size, lymph node metastasis and TNM stage. ORs greater than 1 indicated that decreased LKB1 expression was likely related to poor differentiation, large tumor size (or deep invasion), lymph node metastasis and advanced TNM stage. P-values<0.05 were considered statistically significant.

## Results

### Study Search Information

**Fig 1:**
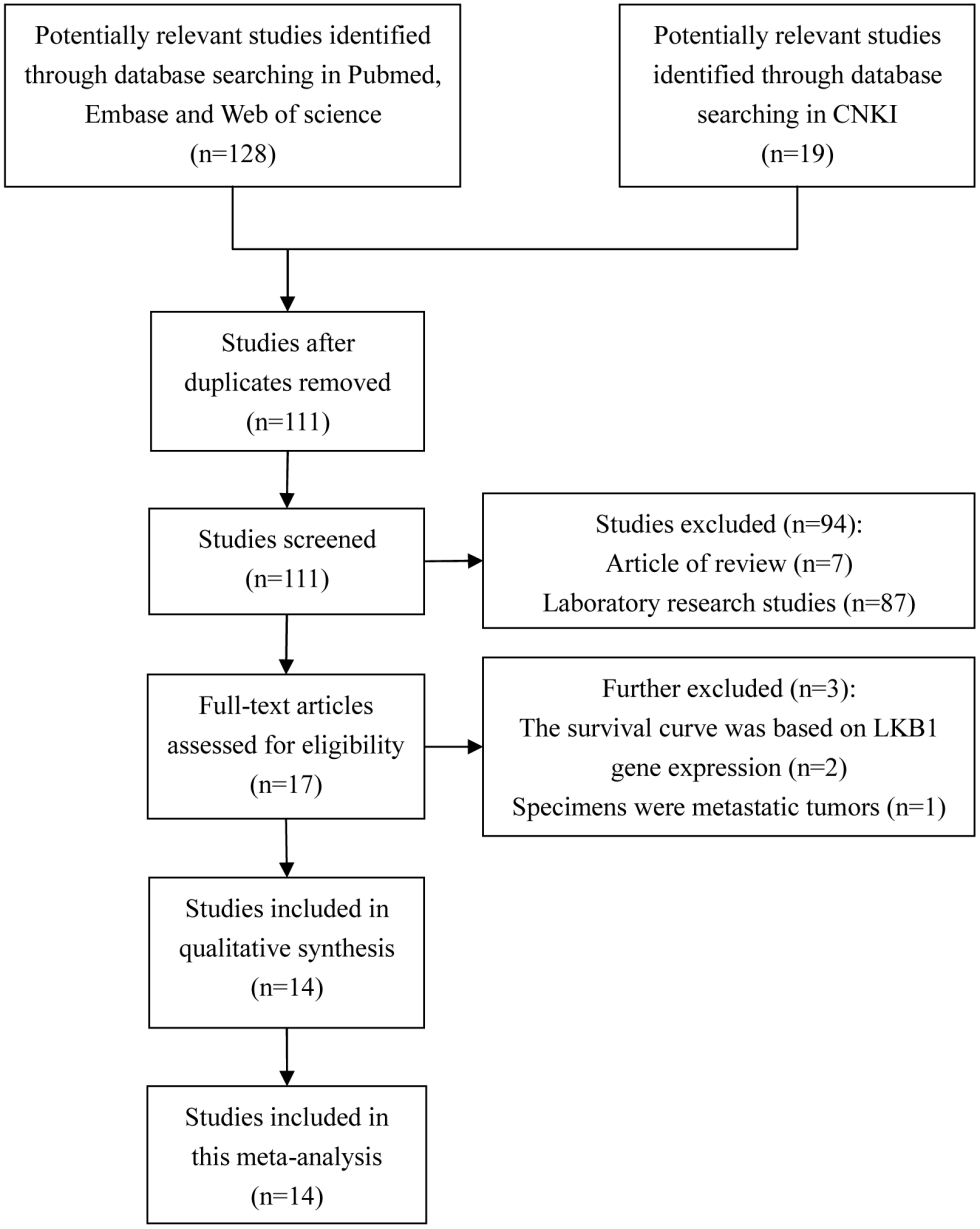
Flow diagram of the selection of eligible studies.

The initial search identified one hundred and eleven potentially relevant titles. A further review of the screening results revealed that seventeen studies were of acceptable relevance for retrieval of the full text. However, two of these studies were excluded because the survival curves were based on LKB1 gene expression [23,24], and one additional study was ruled out because the specimens were metastatic tumors [25]. Ultimately, fourteen studies [11–18,26–31] met the eligibility criteria and were included in the current meta-analysis (Fig 1).

### Description of the Studies

The characteristics of the 14 identified studies are shown in Tables 1 and 2. In total, 1915 patients from five regions (China, Taiwan, the USA, France and the UK) were included in these studies. The solid tumors that were included in this meta-analysis were derived from the following seven cancer types: lung adenocarcinomas (or non-small cell lung cancers), breast carcinomas, gastric cancers, hepatocellular carcinomas, pancreatic cancers (or pancreatic ductal adenocarcinomas), colorectal cancers, and intrahepatic cholangiocarcinomas. The NOS scores of these studies ranged from 5 to 8 (mean: 6.33; S1 and S2 Tables), thus, the studies were of high quality.

**Table 1:** Main characteristics of the eligible studies.

First author	Year	Region	Type of cancer	Number of cases	Median age(range)	Adjuvant therapybefore surgery	Adjuvant therapy after surgery	Follow-up (months)	NOS score
Huang YH [13]	2013	China	Hepatocellular carcinoma	70	57(43–72)	NR	NR	68	7
He TY [14]	2014	Taiwan	Colorectal cancer	158	NR	NR	NR	81	5
Bouchekioua-Bouzaghou K [17]	2014	France	Breast cancer	154	57(27–87)	NR	NR	162	7
Shen Z [11]	2002	China	Breast carcinoma	116	53.7(32–77)	Radiotherapy for 40 cases	Chemotherapy for 56 cases, Hormonal therapy for 43 cases	70	6
Tsai LH [27]	2014	Taiwan	Lung adenocarcinomas	115	NR	None	NR	140	7
Jiang LL [15]	2014	China	Non-small cell lung cancer	142	58.2(31–84)	None	NR	71	7
Yang JY [16]	2015	China	Pancreatic ductaladenocarcinoma	205	NR	None	NR	98	7
Calles A [28]	2015	USA	Lung adenocarcinoma	126	63.5(30–84)	NR	NR	60	7
Wang JH [26]	2015	China	Intrahepatic cholangiocarcinoma	326	NR	NR	NR	99	8
Lee SW [18]	2015	Taiwan	Hepatocellular carcinoma	120	NR	NR	NR	101	7
Morton JP [12]	2010	UK	Pancreatic cancer	106	NR	NR	NR	95	6
Ding XM [29]	2005	China	Lung adenocarcinoma	62	60.5(32–77)	None	Radiotherapy/chemotherapy	80	8
Yang XW [30]	2012	China	Gastric cancer	100	65(31–85)	None	Radiotherapy/chemotherapy	36	7
Huang Y [31]	2014	China	Gastric carcinoma	115	61(37–80)	None	NR	75	6

**Table 2:** LKB1 evaluation and survival data of the selected studies.

First author	Test method	Staining position	Cut-off value	Outcome	Analysis method	HR and 95%CI
Huang YH [13]	IHC	Cytoplasm	Staining index scores of ≤3	OS	UA	3.155(1.603–6.211)
					MA	2.179(1.066–4.444)
				DFS	UA	2.737(1.629–6.271)
He TY [14]	IHC	No specific description	A score equal to or lower than 100	OS	UA	2.364(1.576–4.112)
					MA	3.146(1.876–5.276)
				RFS	UA	2.522(1.701–4.445)
					MA	3.093(1.843–5.191)
Bouchekioua-Bouzaghou K [17]	IHC	Nucleus	Staining intensity recorded as 0	OS	UA	1.417(0.722–2.704)
				DFS	UA	1.279(0.732–2.225)
Bouchekioua-Bouzaghou K [17]	IHC	Cytoplasm	Staining intensity recorded as 0–1	OS	UA	0.418(0.181–0.708)
					MA	0.403(0.199–0.820)
				DFS	UA	0.495(0.249–0.809)
					MA	0.549(0.303–0.990)
Shen Z [11]	WB	Total protein	The bands of the breast cancer tissue in which the quantities were <0.5	OS	UA	3.754(1.899–10.75)
				DFS	UA	2.529(1.383–5.933)
Tsai LH [27]	IHC	No specific description	A score equal to or lower than 100	OS	UA	1.846(1.243–3.202)
					MA	1.868(1.160–3.007)
				RFS	UA	1.828(1.247–3.122)
					MA	1.791(1.132–2.834)
Jiang LL [15]	IHC	Cytoplasm	A score of 0–4	OS	UA	3.226(1.852–5.556)
					MA	2.128(1.136–4.000)
Yang JY [16]	IHC	No specific description	A total score <4	OS	UA	2.278(1.495–3.472)
					MA	1.845(1.189–2.865)
Calles A [28]	IHC	Cytoplasm	No staining	OS	UA	1.44(0.92–2.28)
Wang JH [26]	IHC	Cytoplasm	The staining density was under the median value	OS	UA	1.857(1.498–2.483)
					MA	1.824(1.404–2.377)
Lee SW [18]	IHC	No specific description	The H-score was <the median	OS	UA	0.517(0.284–0.931)
					MA	0.496(0.245–1.047)
				RFS	UA	0.403(0.237–0.624)
					MA	0.333(0.193–0.564)
Morton JP [12]	IHC	Cytoplasm	The histoscore was ≤100	OS	UA	1.877(1.280–4.318)
					MA	1.87(1.09–3.22)
Ding XM [29]	IHC	Both nucleus and cytoplasm	The staining intensity in the neoplasms was lower than that of normal airway epithelium	OS	UA	3.003(2.524–9.635)
Yang XW [30]	IHC	Both nucleus and cytoplasm	The staining intensity in the neoplasms was less than that of normal mucosa	OS	UA	2.558(1.674–4.588)
Huang Y [31]	IHC	Both nucleus and cytoplasm	No staining	OS	UA	2.514(1.026–4.092)

## Decreased LKB1 Expression and OS

**Fig 2:**
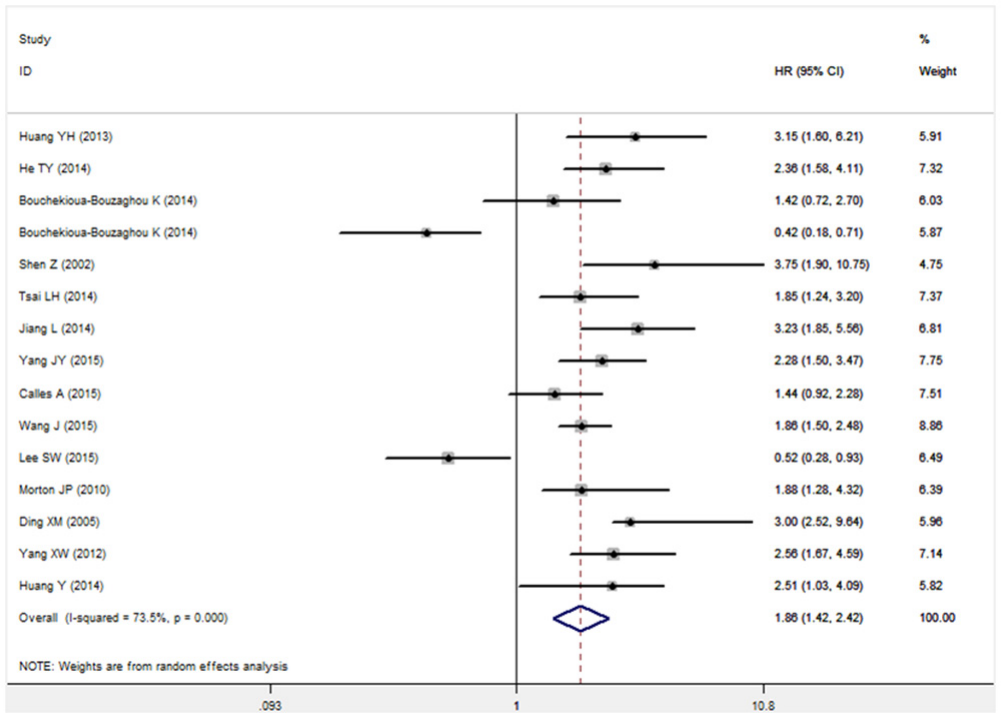
Forest plot describing the association between decreased LKB1 expression and OS.

**Fig 3:**
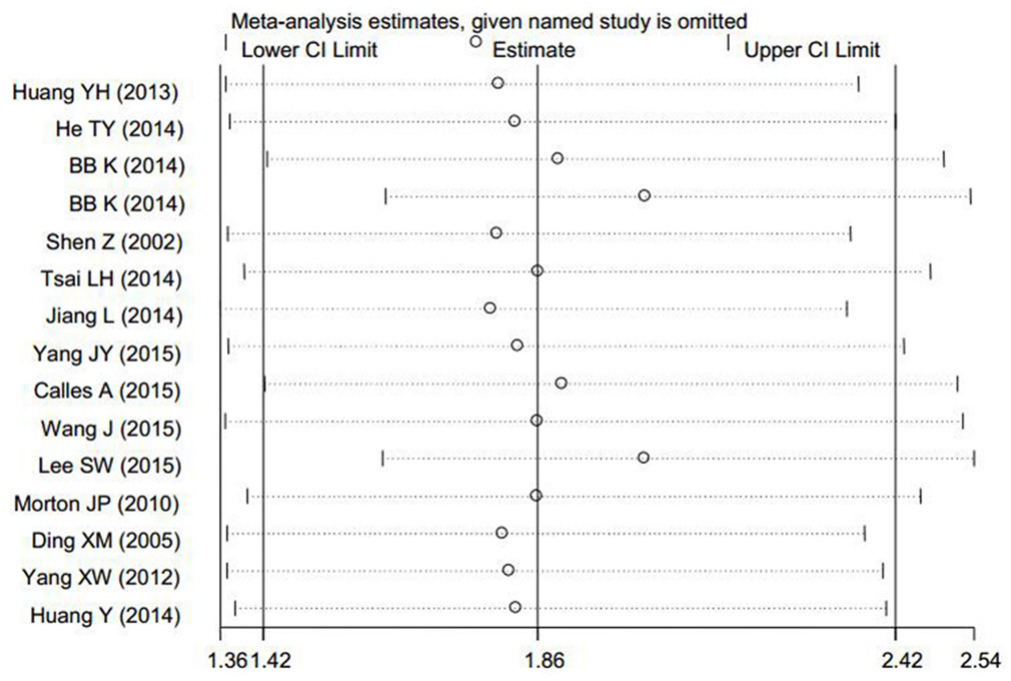
Sensitivity analysis of the OS in the meta-analysis (note: BB K was used as an abbreviation for Bouchekioua-Bouzaghou K because the full name was too long and affected the typesetting of the image).

The pooled HR values revealed that decreased expression of LKB1 protein was significantly associated with OS in relation to solid tumors (HR: 1.86, 95%CI: 1.42–2.42, P<0.001; Fig 2). Additionally, significant heterogeneity (I^2^ = 73.50%, P<0.001) was observed when using a random-effects model to analyze the pooled HR values of the OSs. By successively omitting each study from the aggregated survival meta-analyses, a sensitivity analysis was performed to evaluate the influence of each individual study on the pooled HR. The results revealed that the pooled estimates of the effect of decreased LKB1 expression on the OS of patients with solid tumors did not vary substantially with the exclusion of any individual study, which implies that the results of this meta-analysis are stable (Fig 3).

To minimize heterogeneity, the subgroup analyses were performed according to the multivariate analysis, test method, region, cancer type, and staining position. Both of the subgroup analyses with the multivariate analysis method (HR: 1.55, 95%CI: 1.09–2.21, P = 0.015) and the IHC test method (HR: 1.79, 95%CI: 1.37–2.35, P<0.001) demonstrated that decreased LKB1 expression was evidently related to poor OS in the patients with solid tumors, and the heterogeneities were similar. When stratifying by geographic region, decreased LKB1 expression was significantly associated with poor OS in patients from Asia (HR: 2.18, 95%CI: 1.66–2.86, P<0.001 with less heterogeneity), while the non-Asian subgroup exhibited no association. When grouped according to cancer type, the pooled HRs for lung cancer and other solid tumors were 2.16 (95%CI: 1.47–3.18, P<0.001 with more less heterogeneity) and 1.74 (95%CI: 1.23–2.45, P = 0.002), respectively. In the staining position subgroup, an intimate correlation between decreased LKB1 expression and poor OS was observed in both the cytoplasm studies (HR = 1.69, 95%CI: 1.07–2.68, P = 0.024) and another group (HR = 1.87, 95%CI: 1.30–2.68, P = 0.001), and significant heterogeneity was present (Table 3).

**Table 3:** Associations between decreased LKB1 expression and OS stratified according to the test method, geographic region, cancer type and staining position.

Categories	Subgroups	Reference number	HR (95% CI)	P-Value	Heterogeneity	
					I^2^	P-Value
**Test method**	IHC	[12–18, 26–31]	1.79(1.37–2.35)	<0.001	74.2%	<0.001
**Region**	Asian	[11, 13–16, 27, 29–31]	2.18(1.66–2.86)	<0.001	67.1%	0.001
	Not Asian	[12, 17, 28]	1.15(0.63–2.08)	0.647	75.1%	0.007
**Cancer type**	Lung cancer	[15, 27–29]	2.16(1.47–3.18)	<0.001	52.9%	0.095
	Other types	[11, 13–18, 26, 30, 31]	1.74(1.23–2.45)	0.002	78.1%	<0.001
**Staining position**	Cytoplasm	[12, 13, 15, 17, 26, 28]	1.69(1.07–2.68)	0.024	80.4%	<0.001
	The others	[11, 14, 16–18,27, 29–31]	1.87(1.30–2.68)	0.001	71.4%	0.001

### Decreased LKB1 Expression and RFS/DFS

No significant correlation between decreased LKB1 expression and RFS was observed in the patients with solid tumors in either the univariate group (HR: 1.23, 95%CI: 0.41–3.67) or the multivariate group (HR: 1.23, 95% CI: 0.35–4.33) analysis in the random-effects model with significant heterogeneity (I^2^ = 93.70%, P<0.001; I^2^ = 94.70%, P<0.001, respectively). Moreover, the pooled HR from the univariate analysis method with a random-effects model also indicated that no significant association existed between decreased LKB1 expression and DFS (HR: 1.42, 95% CI: 0.65–3.10) (Table 4).

**Table 4:** Meta-analysis results of decreased LKB1 expression and survival.

Survival data	Analysis method	Reference number	HR (95% CI)	P-value	Heterogeneity	
					I^2^	P-value
**OS**	Univariate analysis	[11–18,26–31]	1.86(1.42–2.42)	<0.001	73.5%	<0.001
	Multivariate analysis	[12–18, 26, 27]	1.55(1.09–2.21)	0.015	76.5%	<0.001
**RFS**	Univariate analysis	[14, 18,27]	1.23(0.41–3.67)	0.709	93.7%	<0.001
	Multivariate analysis	[14, 18,27]	1.23(0.35–4.33)	0.746	94.7%	<0.001
**DFS**	Univariate analysis	[11, 13, 17]	1.42(0.65–3.10)	0.376	83.5%	<0.001

### Correlations of Decreased LKB1 Expression with Clinicopath-Ological Features

The clinical and pathological parameters that were collected from the eligible studies are presented in S3 Table. Meanwhile, Table 5 summarizes the pooled results of the correlations that were identified between decreased LKB1 expression and the clinicopathological features in the patients with solid tumors. No significant correlations of decreased LKB1 expression with age, sex or tumor differentiation were observed. However, the decreased expression of LKB1 was positively associated with tumor size (OR: 1.60, 95%CI: 1.09–2.36, P = 0.017), lymph node metastasis (OR: 2.41, 95%CI: 1.53–3.78, P<0.001) and TNM stage (OR: 3.35, 95%CI: 2.20–5.09, P<0.001).

**Table 5:** Meta-analysis results of the associations of decreased LKB1 expression with clinicopathological parameters.

Clinicopathological parameter	Reference number	Overall OR(95% CI)	P-value	Heterogeneity test(Q, I^2^, P-value)
**Age(≥60 vs <60)**	[15, 29–31]	0.88(0.56–1.39)	0.583	1.74, 0.0%, 0.628
**Sex(male vs female)**	[13–16, 26–30]	0.90(0.71–1.16)	0.418	5.63, 0.0%, 0.689
**Tumor differentiation(poor vs well)**	[11, 13, 15–17, 26, 30]	1.84(0.79–4.30)	0.160	39.24, 82.2%, <0.001
**Tumor size(T3-4 vs T1-2)**	[11, 13, 16, 17, 26, 27, 29–31]	1.60(1.09–2.36)	0.017	17.11, 47.4%, 0.047
**Lymph node metastasis(yes vs no)**	[11, 15–17, 26, 27, 29–31]	2.41(1.53–3.78)	<0.001	29.17, 69.2%, 0.001
**TNM stage(III-IV vs I-II)**	[13–16, 26, 27, 29–31]	3.35(2.20–5.09)	<0.001	18.28, 56.2%, 0.019

### Evaluation of Publication Bias

**Fig 4:**
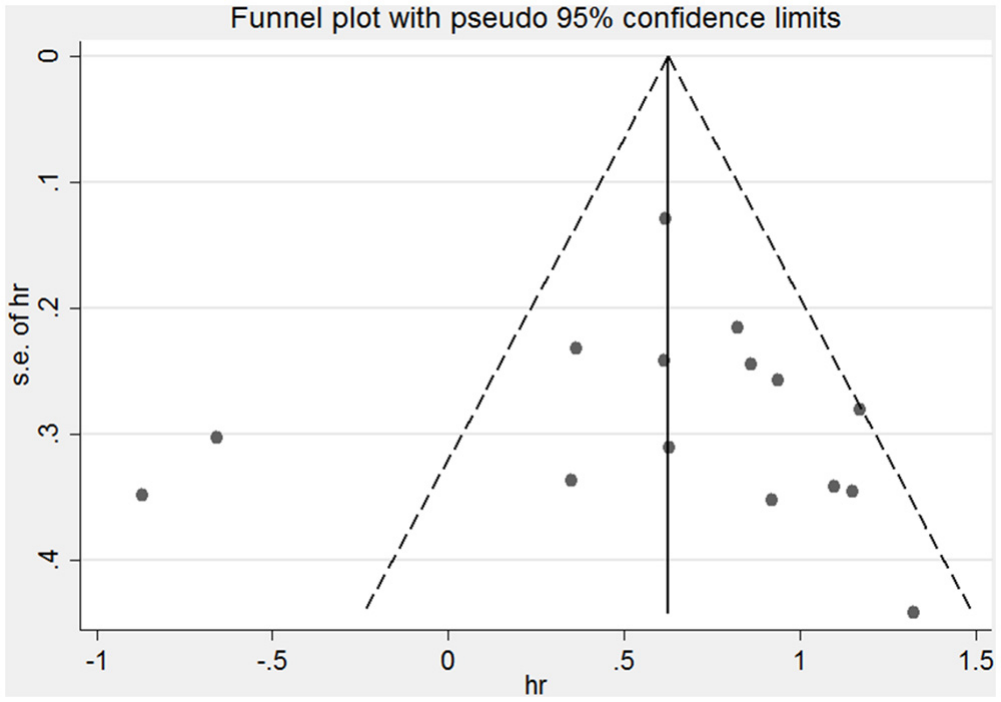
Funnel plot for the assessment of potential publication bias regarding OS in the meta-analysis.

The shape of the funnel plot for the OS appeared to asymmetrical, indicating potential publication bias (Fig 4). However, the Begg’s and Egger’s tests revealed non-significant values (P = 0.322 and 0.928, respectively).

## Discussion

LKB1 is a primary upstream kinase of adenosine monophosphate-activated protein kinase (AMPK) [32] and a required element in cell metabolism for the maintenance of energy homeostasis. LKB1 exerts growth-suppressing effects by activating a group of AMPK-related kinases. The activation of AMPK-related kinases by LKB1 plays vital roles in the maintenance of cell polarity and inhibits the inappropriate expansion of cancer cells. Thus, LKB1 functions as a human tumor suppressor [33,34]. Consequently, decreases in LKB1 can promote cancer progression and are predictive of poor prognoses in patients with cancer [16,35]. However, thus far, no meta-analyses have been performed to evaluate the prognostic value of decreased LKB1 in patients with solid tumors. To the best of our knowledge, this is the first comprehensive meta-analysis of the effects of decreased LKB1 expression on the survival and clinicopathological characteristics of solid tumors.

In this meta-analysis, 14 eligible studies met the inclusion criteria. The data were organized according to OS, RFS and DFS. The combined results demonstrated that decreased LKB1 expression was associated with a poorer OS in solid tumor patients based on a random effects model. The sensitivity analysis revealed that no individual study influenced the overall results, indicating the stability of the pooled results. Additionally, no publication bias was observed. Due to significant heterogeneity between our included studies, we performed further subgroup analyses according to the analysis method, test method, region, cancer type, and staining position. With the exception of non-Asian regions, all of the subgroup analyses indicated that decreased LKB1 expression was associated with poor OS. Regarding the studies that evaluated RFS and DFS, decreased expression of LKB1 was not correlated with either of these factors. However, because the number of articles related to the analyses of RFS and DFS were both no more than 3, these results remain inconclusive and require further investigation. Furthermore, significant associations of decreased LKB1 expression with larger tumor size, lymph node metastasis and higher TNM stage were observed. Therefore, we conclude that decreased LKB1 may serve as a biomarker for poor clinicopathological prognostic factors.

The current analyses have several important implications. First, decreased LKB1 may be a universal poor prognostic marker in solid tumors. In this meta-analysis, we included seven different cancer types, i.e., lung cancer [15,27–29], breast cancer [11,17], gastric cancer [30,31], hepatocellular cancer [13,18], pancreatic cancer [12,16], colorectal cancer [14] and intrahepatic cholangiocarcinoma [26]. The pooled results from these cancer types demonstrated that decreased LKB1 expression was associated with a poor OS and this finding can basically be extended to all solid tumors [22,36–38]. Second, we demonstrated that decreased LKB1 correlated with poor OS in the Asian region but not in the non-Asian region. This discrepancy may have been due to environmental factors that varied in the different regions and different genetic backgrounds [39,40]. Third, decreased LKB1 expression may be a reliable prognostic marker of lung cancer patients with poor OS. Our analysis results revealed that lung cancer patients with decreased expression of LKB1 exhibited significantly poorer OSs. However, because lung cancer is the leading cause of cancer-related death worldwide [41], additional original research regarding the correlation between decreased LKB1 expression and the survival data of patients with lung cancer is needed to verify our results. Fourth, different localizations and specific mutations of LKB1 may alter the association between LKB1 expression and cancer patient survival. LKB1 has different localizations in mammalian cells. The accumulation of LKB1has been detected in both the nuclei and cytoplasm of cells [42,43]. Via the formation of complexes with other proteins [43,44] and under specific conditions [45,46], LKB1 can also translocate from the nucleus to the cytoplasm. Additionally, specific mutations can lead to the loss of the ability of LKB1 to inhibit cell growth and promote cancer progression [47,48]. Thus, the possible mutations in LKB1 maybe among the reasons for the conflicting OS results that included in our meta-analysis.

However, in our meta-analysis, two studies reported inconsistent results that decreased LKB1 might correlate with a favorable survival [17,18], which showing the two obvious outliers on the left of graph in Fig 4. We suspect, aside from the possibility of different localizations and specific mutations of LKB1 discussed above, that the particular molecular phenotypes, such as methylated ERα(metERα) [17] and Skp2-dependent ubiquitination [18], as well as its related mechanisms, of the metERα/Src/PI3K complex [17] and the Skp2-mediated K63-linked polyubiquitination of LKB1 [18] may play primary roles in these contradictory phenomena.

Several limitations should be considered when interpreting our meta-analysis results. One of the main limitations is the significant heterogeneity between the included studies. However, we used a random-effects model with the pooled data. The heterogeneity among these studies could be explained by the different patient characteristics or differences in the specific study designs according to the different tumor types. Another limitation is that some of the survival data were extracted from Kaplan-Meier curves and might have introduced bias. Thus, the present statistics seem to be less reliable than those directly obtained from published studies. One additional limitation is that all of the included studies were designed as retrospective studies, and such studies are more likely to be published if they have positive results than if they have negative results. Therefore, our estimate of the association between decreased LKB1 and outcome may have been overestimated. Finally, the lack of consensus regarding the definition of the cut-off value for decreased LKB1 expression in these included studies might have led to between-study heterogeneity, and we were unable to set a baseline for decreased LKB1 expression which may have resulted in inconsistency.

In summary, this meta-analysis suggested that decreased LKB1 expression significantly contributed to poor OS in solid tumor patients. Decreased LKB1 is also a potential predictive marker for poor clinicopathological prognostic factors in patients with solid tumors. However, further studies related to specific tumor types and perspectives are required to verify the clinical utility of decreased LKB1 in solid tumors.

## Supporting Information

S1 File PRISMA 2009 Checklist. (DOC)

S1 Table Newcastle-Ottawa Scale (NOS) for quality assessment in meta-analysis. (DOCX)

S2 Table Quality assessment of the 14 included studies according to the NOS. (DOCX)

S3 Table Summarized data of clinical and pathological parameters from the eligible studies. (XLSX)
