# Supplementary material for: The Prognostic Value of Decreased LKB1 in Solid Tumors: A Meta-Analysis
Source: PLoS One. 2016 Apr 1;11(4):e0152674. doi: 10.1371/journal.pone.0152674 (PMC4818087; doi:10.1371/journal.pone.0152674)
Supplement: S1 Table — (DOCX) [file pone.0152674.s002.docx]

**S1 Table. Newcastle-Ottawa Scale (NOS) for quality assessment in meta-analysis.**

| **Selection** |
| --- |
| (1) Representativeness of the exposed cohort |
| (a) Truly representative of the cancer patients in the community (1 star) |
| (b) Somewhat representative of the cancer patients in the community (1 star) |
| (c) Selected group of users (e.g., nurses, volunteers) |
| (d) No description of the derivation of the cohort |
| (2) Selection of the non-exposed cohort |
| (a) Drawn from the same community as the exposed cohort (1 star) |
| (b) Drawn from a different source |
| (c) No description of the derivation of the non-exposed cohort |
| (3) Ascertainment of exposure (proof of cancer and LKB1 measurement) |
| (a) Secure record (e.g., surgical records or pathological diagnosis) (1 star) |
| (b) Structured interview (1 star) |
| (c) Written self-report |
| (d) No description |
| (4) Demonstration that outcome of interest was not present at start of study |
| (a) Yes (1 star) |
| (b) No |
| **Comparability** |
| (1) Comparability of cohorts based on the design or analysis |
| (a) The age between exposed cohort and non-exposed cohort had no significant difference (1 star) |
| (b) The sex (or grade, stage, etc.) between exposed cohort and non-exposed cohort had no significant difference (1 star) |
| **Outcome** |
| (1) Assessment of outcome (death or recurrence) |
| (a) Independent blind assessment (1 star) |
| (b) Record linkage (1 star) |
| (c) Self-report |
| (d) No description |
| (2) Was follow-up long enough for outcomes to occur? (death or recurrence) |
| (a) Yes (at least 3 years) (1 star) |
| (b) No |
| (3) Adequacy of follow-up of cohorts |
| (a) Complete follow-up—all subjects accounted for (1 star) |
| (b) Subjects lost to follow-up unlikely to introduce bias—small number lost (less than 25%) or description provided of those lost (1 star) |
| (c) Follow-up rate less than 75% and no description of those lost |
| (d) No statement |
| Note: a maximum of one “star” for each item within the “Selection” and “Outcome” categories, maximum of two “stars” for “Comparability”. |
