# Supplementary material for: The Prognostic Value of Decreased LKB1 in Solid Tumors: A Meta-Analysis
Source: PLoS One. 2016 Apr 1;11(4):e0152674. doi: 10.1371/journal.pone.0152674 (PMC4818087; doi:10.1371/journal.pone.0152674)
Supplement: S2 Table — (DOCX) [file pone.0152674.s003.docx]

**S2 Table. Quality assessment of the 14 included studies according to the NOS.**

| **First author** | **Year** | **Region** | **Type of cancer** | **Selection/4** | **Comparability/2** | **Outcome/3** | **Total score** |
| --- | --- | --- | --- | --- | --- | --- | --- |
| Huang YH [13] | 2013 | China | Hepatocellular carcinoma | 1+1+1+0=3 | 1+1=2 | 1+1+0=2 | 7 |
| He TY [14] | 2014 | Taiwan | Colorectal cancer | 1+1+1+0=3 | 0+0=0 | 1+1+0=2 | 5 |
| Bouchekioua-  Bouzaghou K [17] | 2014 | France | Breast cancer | 1+1+1+0=3 | 1+1=2 | 1+1+0=2 | 7 |
| Shen Z [11] | 2002 | China | Breast carcinoma | 1+1+1+0=3 | 1+0=1 | 1+1+0=2 | 6 |
| Tsai LH [27] | 2014 | Taiwan | Lung adenocarcinomas | 1+1+1+0=3 | 1+1=2 | 1+1+0=2 | 7 |
| Jiang LL [15] | 2014 | China | Non-small cell lung cancer | 1+1+1+0=3 | 1+1=2 | 1+1+0=2 | 7 |
| Yang JY [16] | 2015 | China | Pancreatic ductal  adenocarcinoma | 1+1+1+0=3 | 1+1=2 | 1+1+0=2 | 7 |
| Calles A [28] | 2015 | USA | Lung adenocarcinoma | 1+1+1+0=3 | 1+1=2 | 1+1+0=2 | 7 |
| Wang JH [26] | 2015 | China | Intrahepatic cholangiocarcinoma | 1+1+1+0=3 | 1+1=2 | 1+1+1=3 | 8 |
| Lee SW [18] | 2015 | Taiwan | Hepatocellular carcinoma | 1+1+1+0=3 | 1+1=2 | 1+1+0=2 | 7 |
| Morton JP [12] | 2010 | UK | Pancreaticcancer | 1+1+1+0=3 | 0+0=0 | 1+1+1=3 | 6 |
| Ding XM [29] | 2005 | China | Lung adenocarcinoma | 1+1+1+0=3 | 1+1=2 | 1+1+1=3 | 8 |
| Yang XW [30] | 2012 | China | Gastric cancer | 1+1+1+0=3 | 1+1=2 | 1+1+0=2 | 7 |
| Huang Y [31] | 2014 | China | Gastric carcinoma | 1+1+1+0=3 | 1+0=1 | 1+1+0=2 | 6 |
